# Seed priming versus foliar spraying of biologically synthesized mixed-valence copper oxide(Cu_4_O_3_): improving sunflower growth under salt stress

**DOI:** 10.1186/s12870-026-09334-8

**Published:** 2026-07-01

**Authors:** Eman M.M. Eldebawy, Salwa M. Abdel Rahman, Amel F. Elhusseiny, Eman G. El-Hosary

**Affiliations:** 1 Botany and Microbiology Department, Faculty of Science, Damanhour University, Damanhour, Egypt; 2 Department of Botany and Microbiology, Faculty of Science, Alexandria University, Alexandria, Egypt; 3 Department of chemistry, Faculty of Science, Alexandria University, Alexandria, Egypt

**Keywords:** Salt stress, Sunflower, Green chemistry, Sustainable agriculture, Copper oxide nanoparticles

## Abstract

Climate change is a major global challenge, with soil salinity emerging as a critical factor limiting agricultural productivity. This study reports the biological synthesis of mixed-valence copper oxide (Cu₄O₃) nanoparticles (MVCN) by utilizing *Leucaena leucocephala* seed extract and copper sulfate as precursors, and evaluates their role in mitigating salinity stress in sunflower (*Helianthus annuus* L.) plants. The synthesized MVCN were characterized by EDX, XRD, FTIR, UV–Vis, SEM, TEM, and Zeta potential analyses. FTIR confirmed effective capping by seed extract, while SEM revealed spherical particles (13–18 nm) and TEM indicated diverse morphologies, suggesting anisotropic growth.A pot experiment was conducted under 150 mM NaCl stress, with MVCN applied via seed priming or foliar spraying (100 mg/L). Both treatments enhanced plant growth and biochemical traits, with foliar spraying proving more effective. Growth parameters increased significantly: shoot fresh weight (32%), shoot dry weight (50%), shoot length (56%), root fresh weight (65%), root dry weight (35%), and leaf area (30%). Biochemical improvements included chlorophyll a (49%), soluble sugars (45%), soluble protein (30%), oil content (34%), phenolics (31%), and proline (29%). Conversely, Na content and malondialdehyde (MDA) levels decreased by 34% and 46%, respectively, indicating alleviation of salinity-induceddamage.These findings demonstrate that foliar application of biosynthesized MVCN offers a sustainable and promising strategy to enhance crop resilience under salt stress, contributing to improved agricultural productivity in saline environments.

**Supplementary Information:**

The online version contains supplementary material available at 10.1186/s12870-026-09334-8.

## Introduction

Salt accumulation currently affects about one-fifth of global croplands and nearly half of irrigated farmland, and these proportions are expected to rise further due to climate change, poor irrigation management, and increasing sea levels [1]. Salinity stress disrupts plant function at multiple levels: physiological, biochemical, and molecular. Elevated salinity leads to suppressed growth [2], nutritional deficits [3] and decreased photosynthetic rate [4]. Salinity stress leads to accumulation of ROS [5], which can cause damage to membranes, lipids, protein and, DNA [6].

Sunflower (*Helianthus annuus* L.) is a major global oil crop, and its cultivation relies heavily on water availability, making moisture supply a critical factor in its growth [7]. Salt stress limits water absorption, disrupts nutrient balance [8], and leads to the accumulation of reactive oxygen species [9], which cause cellular damage and ultimately reduce oil content in sunflowers [10].

Recent studies have explored various strategies to mitigate the effects of salinity on sunflower, including sucrose application [11], combined application of gypsum and composted cow dung [9], biochar-based amendments [12], the combined utilization of Bacillus pumilus with biochar as a soil amendment [13], and topical application of salicylic acid and silver nanoparticles [14].

The utilization of nanomaterials in agriculture is very promising due to their effectiveness at low concentrations. This is attributed to the biostimulant properties of nanoparticles when applied at low concentrations [15]. The biological synthesis of nanoparticles represents a sustainable strategy, as it minimizes dependence on toxic chemicals and energy-intensive physical techniques, therefore, conferring considerable ecological advantages [16]. Furthermore, nanoparticles produced via biological routes demonstrate significantly reduced cytotoxic and phytotoxic effects compared to those generated through conventional chemical and physical methods, which means they are safe and can be widely used [17].

Significant attention has been garnered to copper oxide nanoparticles not only for their versatile applications but mainly for their cost-effectiveness, easiness of accessibility, and their similarity to the properties of noble metal [18, 19] CuO (tenorite), Cu_2_O (cuprite), and Cu_4_O_3_ (paramelaconite) are the three phases of oxide that are produced when copper, a multivalent metal, reacts easily with oxygen. Of the three phases, Cu_4_O_3_ is the most uncommon. It is a metastable copper oxide with an atomic ratio of 1.33 that includes both Cu(II) and Cu(I) oxidation states [20]. Because it is exceedingly difficult to stable the Cu^+^ and Cu^2+^ ions concurrently, the synthesis of Cu_4_O_3_ using traditional chemical methods is quite challenging [21]. However, because of its environmental friendliness, the green synthesis route, which uses bioresources like plants, has gained popularity. Additionally, green methods provide safer and more environmentally friendly ways to form nanoparticles with a variety of shapes, sizes, compositions, and physicochemical properties [22].

Copper-based nanoparticles (Cu-NPs) have appeared as innovative nanoagrochemical agents, demonstrating the capacity to modulate a wide range of plant stress-responsive signaling pathways [23]. It has been verified that copper oxide nanoparticles significantly diminish the negative effects of salt in a variety of agricultural crops. including wheat [24], barley [25], tomato [26], and maize [27], whether applied through seed priming or foliar spraying. Copper is an essential micronutrient in plants, functioning as a redox-active transition metal capable of cycling between the Cu⁺ and Cu²⁺ states. This redox versatility enables copper to play a central role as both a structural element and a catalytic cofactor in several plant enzymes such as cytochrome c oxidase, superoxide dismutase, and plastocyanin supporting diverse biochemical and physiological processes including photosynthesis, respiration and enzymatic antioxidant system [28, 29]. At the nanoscale, copper provides distinct advantages offering enhanced bioavailability, controlled nutrient release, and precise delivery at the cellular level compared to conventional fertilizers [30].

While copper oxide nanoparticles have been produced by utilizing plant extracts in previous studies 9 [20, 31], the utilization of Leucaena leucocephala seed extract for the biosynthesis of Cu_4_O_3_ NPs remains largely uninvestigated. *L. leucocephala* seeds’ diverse phytochemical composition, which includes flavonoids and phenolic constituents [32], fatty acids [33], and Polysaccharides [34] forms a distinctive sequence of reducing and stabilizing agents that may increase the biological properties of the synthesized nanoparticles.

The goal of this research was to synthesize copper nanoparticles using biological approache, and to evaluate their potential in helping sunflower plants withstand the harmful effects of salt stress by evaluating changes in physiological and biochemical processes. Understanding how copper oxide nanoparticles influence sunflower growth and stress physiology could provide valuable insights for improving crop resilience under saline conditions.

## Materials and methods

### Biological synthesis of copperoxide nanoparticles

*Leucaena leucocephala* plants were collected from El-Beheira, Egypt. The herbarium sheet has been deposited in the Tanta University Herbarium (TANE) under Accession No. 13,456. The identification was revised, and the voucher number assigned by the Herbarium Manager, Prof. Dr. Dalia A. Ahmed (Professor of Plant Ecology), together with Dr. Salma K. Shaltout (Botany and Microbiology Department, Faculty of Science, Tanta University).The seeds were washed and air-dried, then grinded to powder. 25 g of seed powder was soaked in 500 mL distilled water and left it for twenty four hours under room conditions for the preparation of plant extract, then was filtered using filter paper (Whatman no. 1) and preserved in a refrigerator until used. Copper sulfate, was used as a precursor for the synthesis of copper oxide nanoparticles. A quantity of 0.428 g of Copper sulfate was dissolved in 500 ml to prepare 3mM CuSO_4_.5H_2_O. The synthesis of copper oxide based nanoparticles was achieved by mixing the (1: 2 v/v) of metal solution and L. leucocephala seed extract. : which frequently resulted in a color change from blue to green. The green color is a key indicator of successful, environmentally friendly biosynthesis of copper-based nanoparticles. The mixture was heated at 40 Cº for one hour and left overnight at room temperature. To harvest the synthesized nanoparticles, the mixture was centrifuged at 5000 rpm for 20 min. The observed precipitate was washed with distilled water more than one time to remove any impurities and centrifuged again, then washed with 70% ethyl alcohol, then centrifuged, and the resulting precipitate was dried at 30 °C [35].

### Physical and chemical characterization of the biosynthesized MVCN

Methods and measurements needed to characterize the green synthesized cupperoxide nanoparticles including Fourier transform infrared spectroscopy, UV–Visible spectroscopy, Energy-dispersive X-ray, Scanning electron microscopy, Transmission electron microscopy, X-ray diffraction and Zeta potential measurement were described in the Supplementary Information (SI ).

### Invistigation of the effect of the biologically synthesized MVCN on growth parameters of Helianthus annuus under salt stress

#### Seed priming

*Helianthus annuus* Seeds (Giza 120) were obtained from the Agricultural Research Center, El-Dokki, Giza, Egypt. Sunflower seeds were surface-sterilized by immerseion in a 0.1% sodium hypochlorite solution for 3 min, followed by repeated washing with distilled water. The sterilized seeds were then soaked for 6 h [36] in 100 mL of an aqueous MVCN solution at concentration 100 mg/L.

#### Foliar spraying

*Helianthus annuus* Seedlings were sprayed with aqueous MVCN solution at concentration 100 mg/L, once per week, with a range of 300 mL/pot [15].

#### Experimental conditions

Uniform seeds (10 seeds per pot) were sown in pots (15 cm diameter × 10 cm height). The experimental design consisted of the following treatments: C = control (irrigating plants with fresh water ); T1 = seed priming in 100 mg/L of aqueous solution of MVCN. ; T2 = foliar spraying with 100 mg/L of aqueous solution of MVCN ; T3 = salt stress (150 mM NaCl); T4 = salt stress + seed priming in 100 mg/L of aqueous solution of MVCN; T5 = salt stress+ foliar spraying with 100 mg/L of aqueous solution of MVCN. The pots were maintained under natural conditions, with 16 h of light and 8 h of darkness, temperatures of 30 ± 2 °C in the glass house of the Faculty of of Science, Damanhour University. Each treatment consisted of three replications that were randomly distributed.

#### Growth parameters

Shoot and root fresh and dry weight, shoot length,, and leaf area was estimated using the equation by Milford et al. [37].

#### TEM

The second leaf fragments from four treatments (C, T3, T4, and T5) were prepared as stated by the method of [38]. Leaf ultra-thin sections were obtained using a diamond knife on an ultramicrotome (Leica EM UC6, Germany) and subsequently mounted onto copper grids with a 300-square mesh.The cell ultrastructure was seen and photographed using a transmission electron microscope at various magnifications.

#### Determination of photosynthetic pigments

The photosynthetic pigments chlorophyll a, b (chl.a, chl.b) and carotenoids (carot.) were determined according to the method described by Inskeep and Bloom [39]. The known weight of the dissected plant leaves (0.035 gm) was incubated in 6 mL N, N-dimethyl formamide (DMF) reagent and kept at 4 °C for 24 h in the dark. The extract-containing pigments were decanted, and the absorbance was measured at the following wavelengths: 647, 665, and 453 nm using a spectrophotometer (JENWAY, 6305, UK). The formula and extinction coefficients used for the determination of chl. a, chl.b, and carotenoids were:

$$\text{Chl. a}={12.70}\;\mathrm{A665}-\text{2.79 A647}$$

$$\text{Chl. b}={20.70}\;\mathrm{A647}-\text{4.62 A665}$$

$$\mathrm{Carotenoids}\;=\text{4.2 A453}-\left(0.0264\;\text{chl. a}+.426\;\text{chl. b}\right)$$

The values were then expressed as mg /g FW.

#### Determination of soluble sugars and soluble protein

Soluble sugars were determined by the method described by Dubois et al. [40]. To 0.5 mL of dry leaf water extract (0.056 gm in 15 mL of distilled water), 1 mL of 5% phenol, followed by 5 mL of concentrated H_2_SO_4_, was added rapidly. The tubes were allowed to stand for 10 min, and absorbance was recorded at 490 nm. A calibrated curve using pure glucose was made from the amount of sugar that was calculated as mg /g DW.

Protein content was determined using the method described by Hartree [41]. Briefly, 0.5 mL of the dry powder leaf water extract was mixed with 0.9 mL of Reagent A (prepared by dissolving 2 g of potassium sodium tartrate and 100 g of sodium carbonate in 1 L of 0.1 N sodium hydroxide). The mixture was incubated in a water bath at 50 °C for 10 min. After cooling to room temperature, 0.1 mL of Reagent B (containing 2 g of potassium sodium tartrate and 1 g of CuSO₄·5 H₂O dissolved in 100 mL of 0.1 N sodium hydroxide) was added and thoroughly mixed. The tubes were then left to stand for 10 min. Subsequently, 3 mL of Reagent C (a 1:10 dilution of Folin–Ciocalteu reagent with distilled water) was added rapidly with vortex mixing. The tubes were incubated again at 50 °C for 10 min. After cooling, absorbance was measured at 650 nm, and protein concentration was determined using a standard curve prepared with bovine serum albumin.

#### Determination of proline and glycinebetaine content

Free proline was extracted according to the method described by El-Sharkawi and Michel [42]. 0.3 of fresh leaf was ground with 10 ml of 3% sulfosalicylic acid and centrifuged at 7000 rpm for 20 min. The supernatant was decanted and stored. The precipitated residual material in the centrifuge tube was transferred back to the mortar and extracted again with sulfosalicylic acid, then centrifuged. The combined extracts were adjusted to a known volume with 3% sulfosalicylic acid. Free proline was carried out using the acid ninhydrin method described by Bates et al. [43]. One ml from the extract was mixed with 2 ml of the freshly prepared acid ninhydrin reagent (Dissolve 1.25 g ninhydrin in 30 ml of warm glacial acetic acid and 20 ml 6 M phosphoric acid) plus 2 ml glacial acetic acid in a test tube and kept for one hour in a boiling water bath, then the reaction was terminated by cooling the mixture in an ice bath. Four mL of toluene were added and mixed vigorously using a vortex. The colored toluene layer was aspirated in a separate test tube and warmed up to room temperature, and then its absorbance was read at 520 nm using toluene as a blank. The proline concentration was determined from a standard curve and expressed as µg /g FW.

Grieve and Grattan’s method [44] was used to determine the glycine betaine content. First, a test tube containing 0.25 g of dried leaf tissue and 10 mL of distilled water was shaken for 24 h. A test tube containing 0.25 mL of the filtrate and 0.25 mL of 2 N sulfuric acid was put in an ice bath for an hour after the solution had been filtered through Whatman filter paper. After adding 0.2 mL of potassium iodide (KI-I2) reagent, the mixture was refrigerated for 16 h at 4 °C. The materials were then centrifuged for 25 min at 6000 rpm, producing two phases. The lower phase was treated with 6 mL of dichloroethane and repeatedly vortexed after the upper phase was discarded. Lastly, a spectrophotometer was used to measure the absorbance of the final samples at 365 nm. The leaf samples’ glycine betaine concentration was determined and expressed as µmol/ g FW.

#### Determination of phenolic content

Extraction of the total phenolic compounds (0.12 g dry powder leaf) was performed with 5 mL of 80% methanol for 24 h at 4 °C. Samples were centrifuged for 10 min at 5000 rpm, and the liquid phase was collected. The procedure was repeated three times, and the pooled supernatants were used for the analysis [45]. The total phenolic content of the leaf extract was determined using a modified Folin-Ciocalteu spectrophotometric method [46]. An aliquot of plant extract was added to 1.5 mL of distilled water, and 100 µL of Folin-Ciocalteau reagent (Sigma) was added. The reaction mixture was shaken and allowed to stand for 5 min before the addition of 300 µL of 20% Na_2_CO_3_. After 20 min at 40 °C, the absorbance was measured at 765 nm against each blank. The phenolic content was calculated as µmol GAE/g DW from the standard curve of different concentrations of gallic acid.

#### Determination of oil content

Total oil content was determined using Soxhlet extraction according to the official methods of the American Oil Chemists’ Society [47]. Briefly, a known weight of dried and powdered plant leaf was extracted with petroleum ether (40–60 °C) for 6 h using a Soxhlet apparatus. After extraction, the solvent evaporated, and the extracted oil was dried and weighed. The oil content was calculated as a percentage of dry weight.

#### Determination of Na + and K+ ions

Dried plant samples were oven-dried, ground, and subjected to wet acid digestion. Briefly, 0.5 g of the dried sample was digested using concentrated sulfuric acid and hydrogen peroxide until a clear solution was obtained. The digest was filtered and diluted with distilled water to a known volume. Sodium (Na) and potassium (K) concentrations were determined using a Perkin-Elmer flame photometer according to Jones and Case [48] and Page et al. [49].

#### Determination of lipid peroxidation

The level of lipid peroxidation was measured according to the thiobarbituric acid (TBA) method. Fresh plant materials (300 mg) were homogenized in 10 ml 0.1% (w/v) trichloroacetic acid (TCA) solution on ice. The homogenates were centrifuged at 15.000 rpm for 5 min at 4 °C, and the supernatants were collected in clean test tubes. One ml of 20% (w/v) TCA containing 0.5% (w/v) TBA solution was added to a 1 ml aliquot of plant extract. The mixture was heated at 95 °C for 30 min and then quickly cooled in an ice bath and centrifuged at 15,000 rpm for 10 min. The optical density of the supernatant was taken at 532 nm, followed by correction for the non-specific absorbance at 600 nm. The absorbance at 600 nm was subtracted from the absorbance at 532 nm, and the MDA content was determined using the extinction coefficient of 155 mM^− 1^ cm^− 1^ [50] and expressed as µmol/g Fw.

### Data analysis

All treatments were conducted in triplicate. The data were analyzed using analysis of variance (ANOVA), and differences among means were evaluated with Tukey’s test at a significance threshold of *p* < 0.05 [51]. Correlation analysis was performed with OriginPro 2026 software.

## Results

### Characterization of the the biosynthesized MVCN

**Fig. 1:**
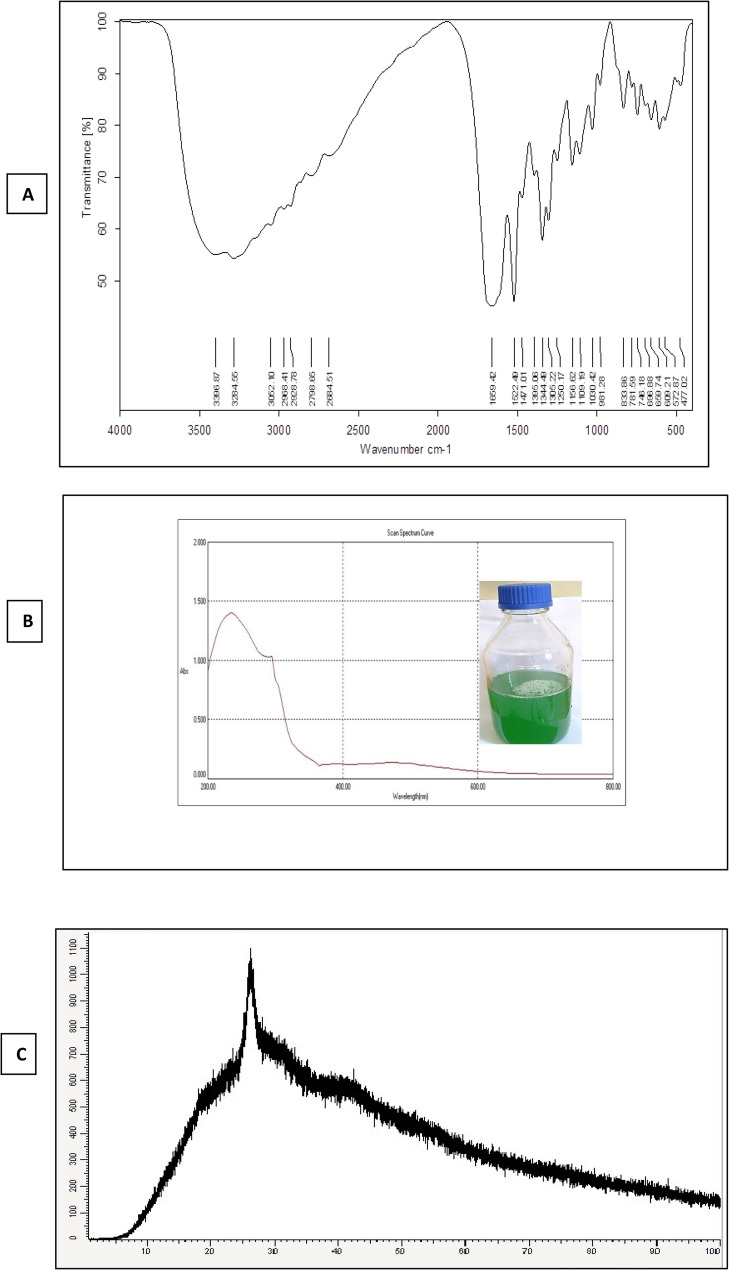
**A** Infrared spectra, (**B**) UV–Vis absorption spectrum, (**C**) XRD pattern of the biologically synthesized MVCN

The FTIR spectra of the biologically synthesized MVCN are presented in (Fig. 1A). FTIR spectroscopy identified the bioactive components’ functional groups that aided in the stability, capping, and reduction of the biologically synthesized MVCN prepared by Leucaena leucocephala seed extract. The UV–visible spectrum of the biosynthesized MVCN was recorded in water at the wavelength range of 200–800 nm. As illustrated in Fig. 1B, the electronic absorption spectrum exhibits absorption bands at 235 ,475 nm and a shoulder at 295 nm. The XRD pattern of the biosynthesized MVCN exhibited broad diffraction peaks (Fig. 1C), which generally attributed to particle size effect. The crystal planes and the 2θ values agreed well with the standard PDF card No. 33–480. It belongs to tetragonal copper oxide paramelaconite. These results clearly show that the the synthesized mixed-valence copper oxide was successfully formed.

**Fig. 2:**
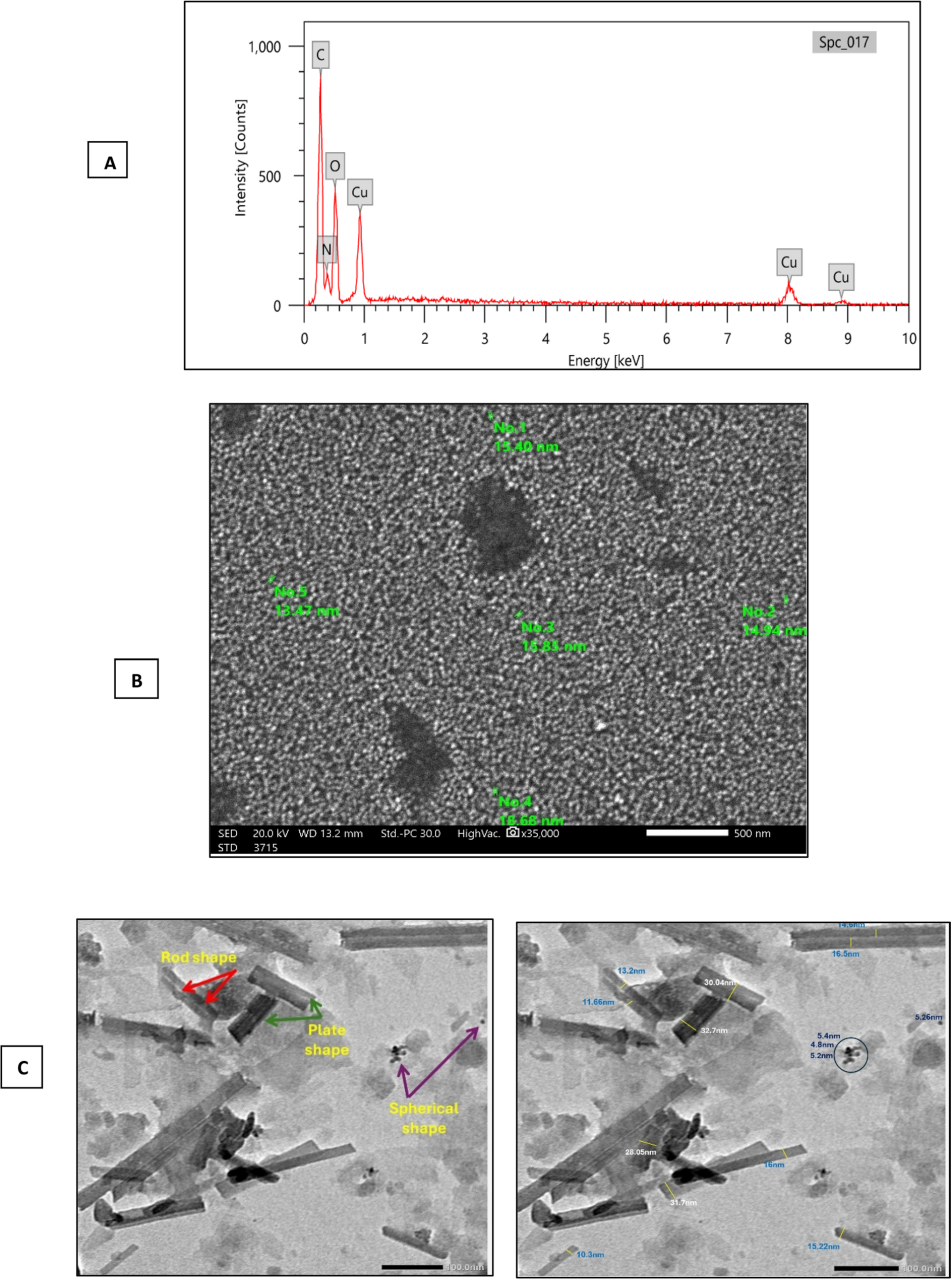
**A** EDS spectra, (**B**) SEM images, (**C**) TEM images of the biologically synthesized MVCN

The EDS results reveal the composition of elements present in the synthesized Cu_4_O_3_ nanoparticles (Fig. 2A). The detected elements include carbon, oxygen, nitrogen, and copper. Among these, carbon exhibited the highest weight% (wt%) of 38.64%.

The average diameter of the synthesized Cu_4_O_3_ nanoparticles was determined from SEM pictures was chosen arbitrarily (Fig. 5). The average diameter was 14.91 ± 0.894 nm, and the mixed valence oxide was composed of spherical nanoparticles.

Figure 2C demonstrates the TEM pictures of the synthetic Cu_4_O_3_ nanoparticles. The transmission electron microscopy assisted in comprehension the particle size-shape, where as three different shaped particles, rod, plate and spherical shape were seen from the TEM pictures (Fig. 2C) and the particles were strongly agglomerated.

### Effect of the biologically synthesized MVCN on growth parameters and chlorophyll content of sunflower under salt stress

**Fig. 3:**
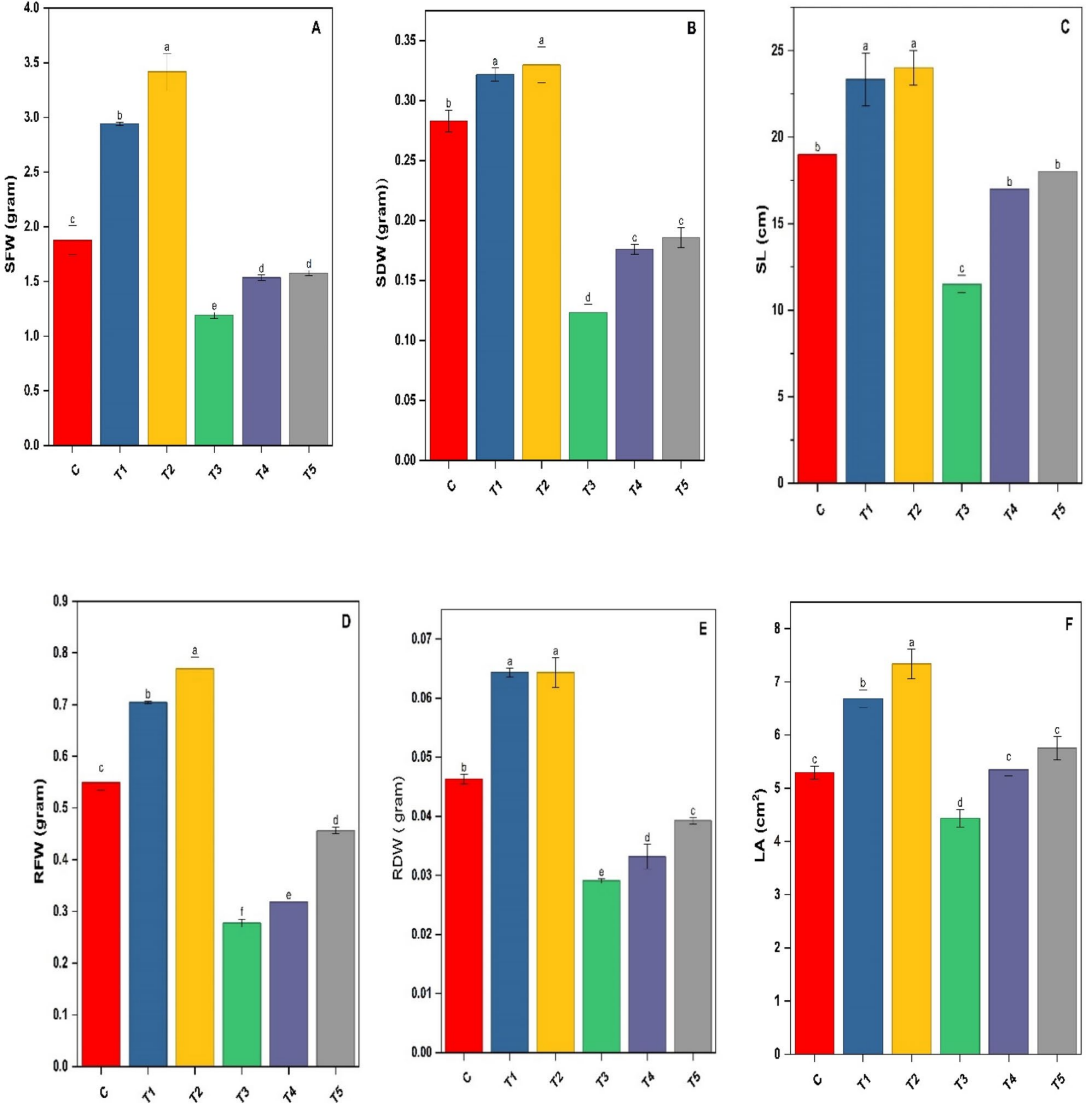
Effect of the biologically synthesized MVCN on growth parameters of sunflower under salt stress. **A** shoot fresh weight, (**B**) shoot dry weight, (**C**) shoot length, (**D**) root freh weight, (**E**) root dry weight, (**F**) leaf area, C = control; T1 = seed priming in 100 mg/L of aqueous solution of MVCN. ; T2 = foliar spraying with 100 mg/L of aqueous solution of MVCN ; T3 = salt stress (150 mM NaCl); T4 = salt stress + seed priming in 100 mg/L of aqueous solution of MVCN; T5 = salt stress+ foliar spraying with 100 mg/L of aqueous solution of MVCN. Different letters on the bars are significantly different as evaluated by Tukey test at a significant level of *p* < 0.05

Generally, the application of the biologically synthesized MVCN resulted in improved growth of sunflower plants, whether through foliar spraying or seed soaking, with foliar spraying producing more effective results than seed soaking (Fig. 3). Salt stress significantly impacted the growth of sunflower plants, reducing shoot fresh weight, shoot dry weight, shoot length, root freh weight, root dry weight and leaf area, by 36%, 56%, 39, 50%, 37%, and 16%, respectively, compared to control plants. However, foliar spraying with aqueous solution of the biologically synthesized MVCN under salt stress improved these parameters by 32%, 50%, 56%, 65%, 35%, and 30% respectively, compared to non-treated plants.

**Fig. 4:**
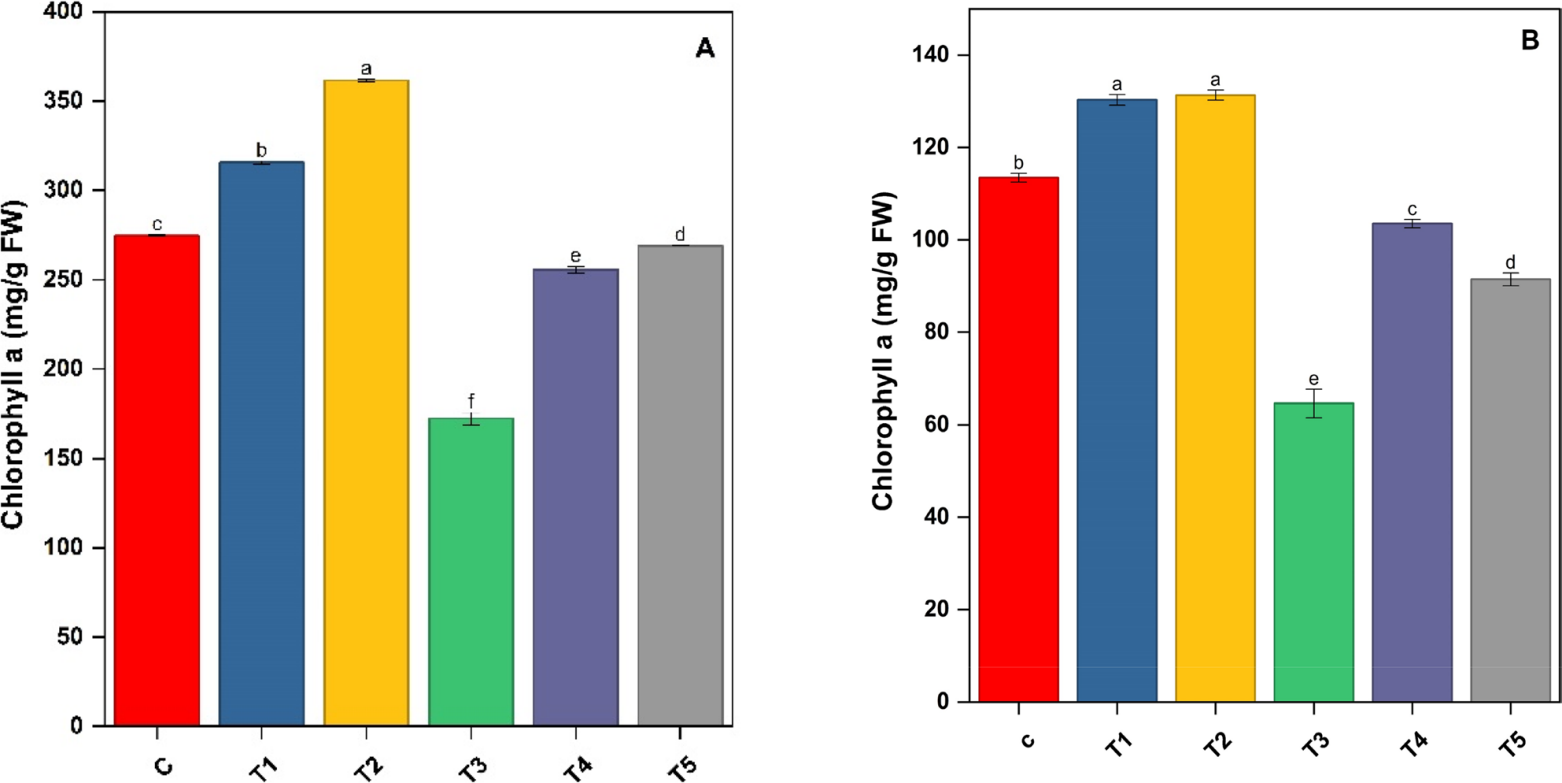
Effect of the biologically synthesized MVCN on photosynthetic pigments of sunflower under salt stress. **A** chlorophyll a content, (**B**) chlorophyll b content, C = control; T1 = seed priming in 100 mg/L of aqueous solution of MVCN. ; T2 = foliar spraying with 100 mg/L of aqueous solution of MVCN ; T3 = salt stress (150 mM NaCl); T4 = salt stress + seed priming in 100 mg/L of aqueous solution of MVCN; T5 = salt stress+ foliar spraying with 100 mg/L of aqueous solution of MVCN. Different letters on the bars are significantly different as evaluated by Tukey test at a significant level of *p* < 0.05

The effect of the biologically synthesized MVCN and salt stress on chlorophyll content of sunflower is presented in Fig. 4. Chlorophyll a decreased by 37% in salt-stressed plants compared to the control ones. However, foliar spraying with aqueous solution of the biologically synthesized MVCN substantially improved the content of chlorophyll a by 49% compared to non-treated plants under saltstree stress. Regarding the content of chlorophyll b, nanotreament (T5) resulted in a 42% increase under salt stress compared to the non-treated plants. Furthermore, nanotreatments (T5) led to an increase in chlorophyll a and b content by 32% and 16%, respectively, in plants that were not exposed to salt stress.

### Effect of the biologically synthesized MVCN on leaf ultrastructure of *sunflower* under salt stress

**Fig. 5:**
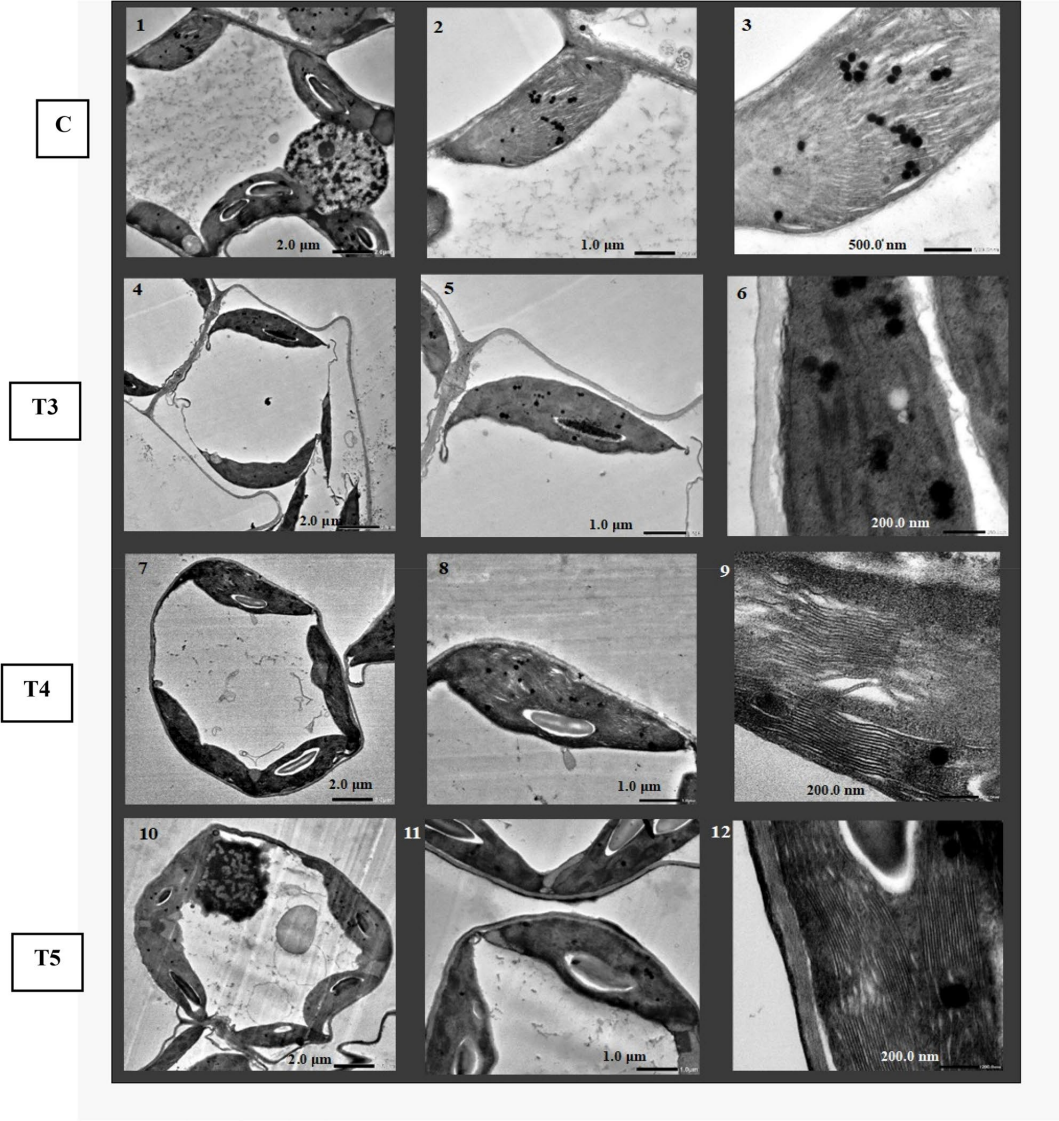
Effect of the biologically synthesized MVCN on leaf ultrastructure of sunflower under salt stress.C = control; T3 = salt stress (150 mM NaCl); T4 = salt stress + seed priming in 100 mg/L of aqueous solution of MVCN; T5 = salt stress+ foliar spraying with 100 mg/L of aqueous solution of MVCN

A TEM micrograph of sunflower leaves is presented in Fig. 4. The mesophyll cells of control sunflower plants exhibit a well-defined cell wall, continuous cell membrane, and a well-developed nuclear envelope (Figs. 5C). In contrast, salt-stressed plants display noticeable alterations in their ultrastructure (Figs. 5-T3). The leaves of salt-stressed plants contain distorted chloroplasts and the internal membrane system of thylakoids (grana and stroma) becomes disorganized. However, the application of the biological synthesized MVCN to salt-stressed plants results in marked improvements in leaf ultrastructure, with chloroplasts regaining their typical elliptical shape and thylakoid membranes appearing well organized, regardless of whether the treatment is applied through seed soaking or foliar spraying (Figs. 5-T4 &T5).

### Effect of the biologically synthesized MVCN on soluble sugars, soluble protein and oil content of sunflower under salt stress stress

**Fig. 6:**
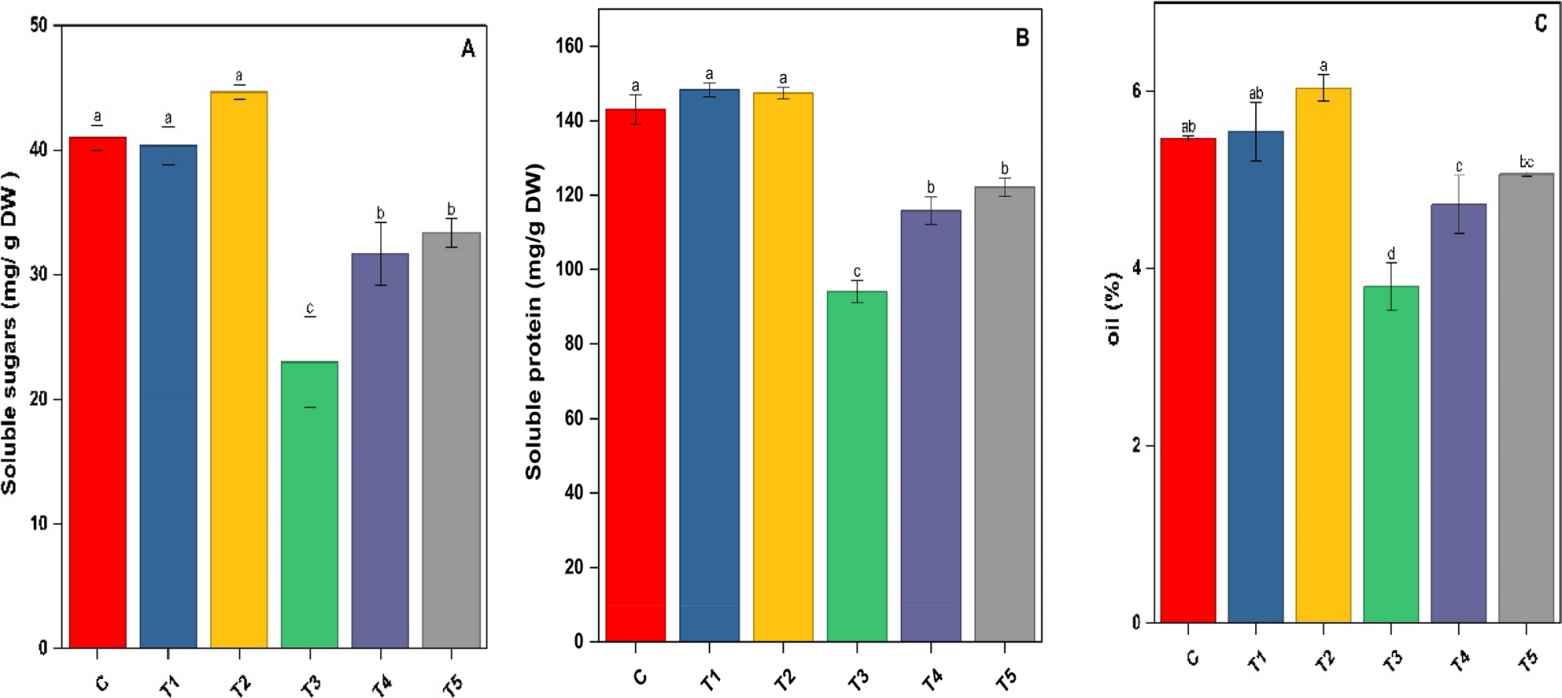
Effect of the biologically synthesized MVCN on soluble sugars, soluble protein and oil content of sunflower under salt stress. **A** soluble sugars, (**B**) soluble protein, (**C**) oil content, C = control; T1 = seed priming in 100 mg/L of aqueous solution of MVCN. ; T2 = foliar spraying with 100 mg/L of aqueous solution of MVCN ; T3 = salt stress (150 mM NaCl); T4 = salt stress + seed priming in 100 mg/L of aqueous solution of MVCN; T5 = salt stress+ foliar spraying with 100 mg/L of aqueous solution of MVCN. Different letters on the bars are significantly different as evaluated by Tukey test at a significant level of *p* < 0.05

Salt stress significantly reduced soluble sugars and soluble protein contents in sunflower ( Fig. 6A&B), whereas nanotreatment (T5) caused a significant increase in the content of soluble sugars and soluble protein by 45% and 30%, respectively, compared to the non-treated plants (T3). Salt stress caused a 30% decrease in oil content compared to the control plants (C), whereas nano-treatment (T5) led to a 34% increase in oil content compared to the non-treated plants (Fig. 6C). In addition, nano-treatment, whether through seed soaking or foliar spraying, led to a significant increase in oil percentage in plants not exposed to salinity.

### Effect of the biologically synthesized MVCN on proline, glycine betaine and phenolic content of suflower under salt stress stress

**Fig. 7:**
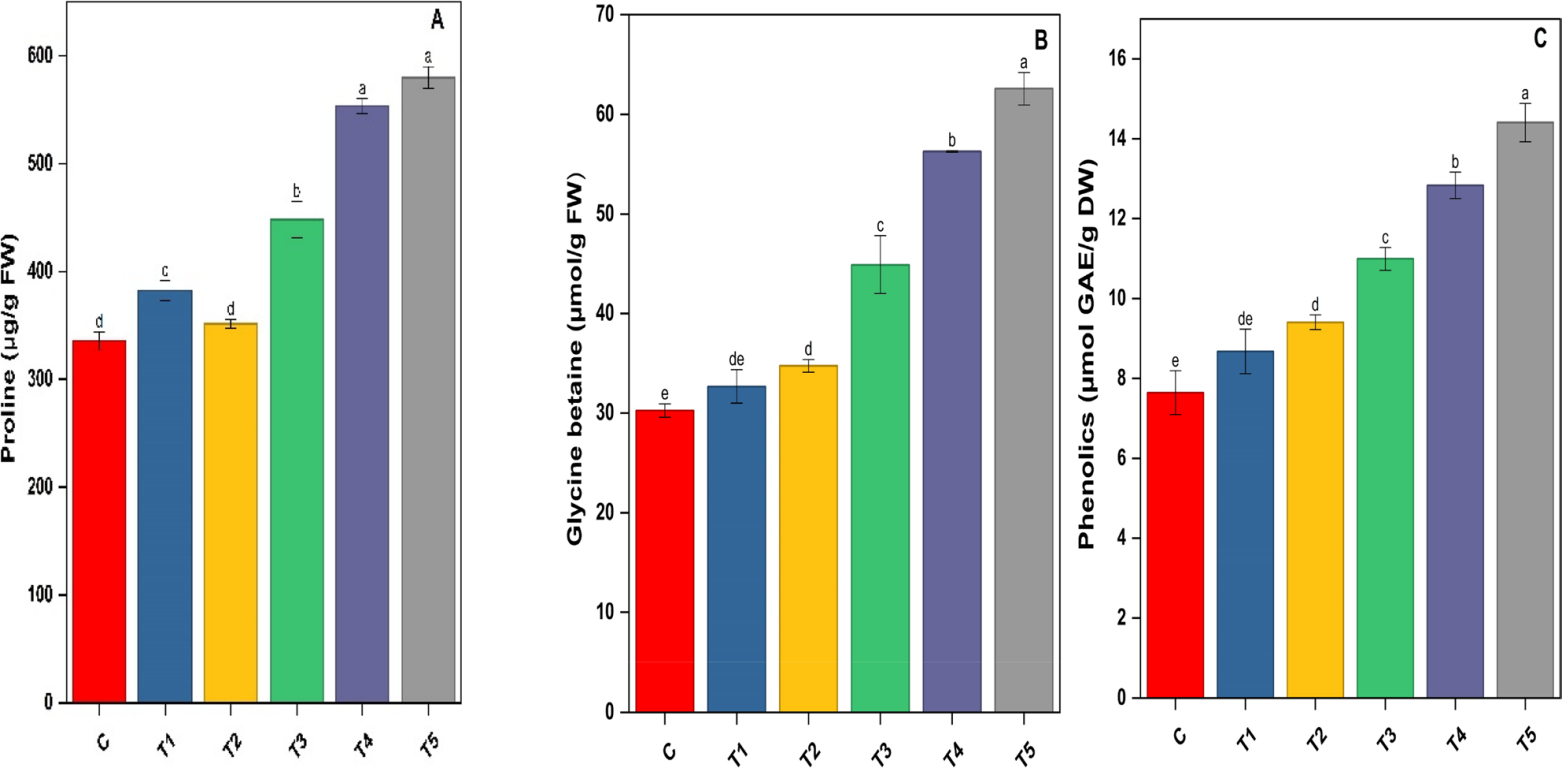
Effect of the biologically synthesized MVCN on proline, glycine betaine and phenolic content of sunflower under salt stress. **A** proline, (**B**) glycine betaine, (**C**) phenolic content, C = control; T1 = seed priming in 100 mg/L of aqueous solution of MVCN. ; T2 = foliar spraying with 100 mg/L of aqueous solution of MVCN ; T3 = salt stress (150 mM NaCl); T4 = salt stress + seed priming in 100 mg/L of aqueous solution of MVCN; T5 = salt stress+ foliar spraying with 100 mg/L of aqueous solution of MVCN. Different letters on the bars are significantly different as evaluated by Tukey test at a significant level of *p* < 0.05

Exposure of plants to salt stress led to increases of 33%, 48%, and 44% in proline, glycine betaine, and phenolic content, respectively (Fig. 7). However, nano-treatment (T5) under salt stress resulted in increases of 29%, 39%, and 31% in proline, glycine betaine, and phenolic content, respectively, compared to non-treated plants.

### Effect of the biologically synthesized MVCN on sodium, Potassium, potassium sodium ratio and MDA content of sunflower under salt stress stress

**Fig. 8:**
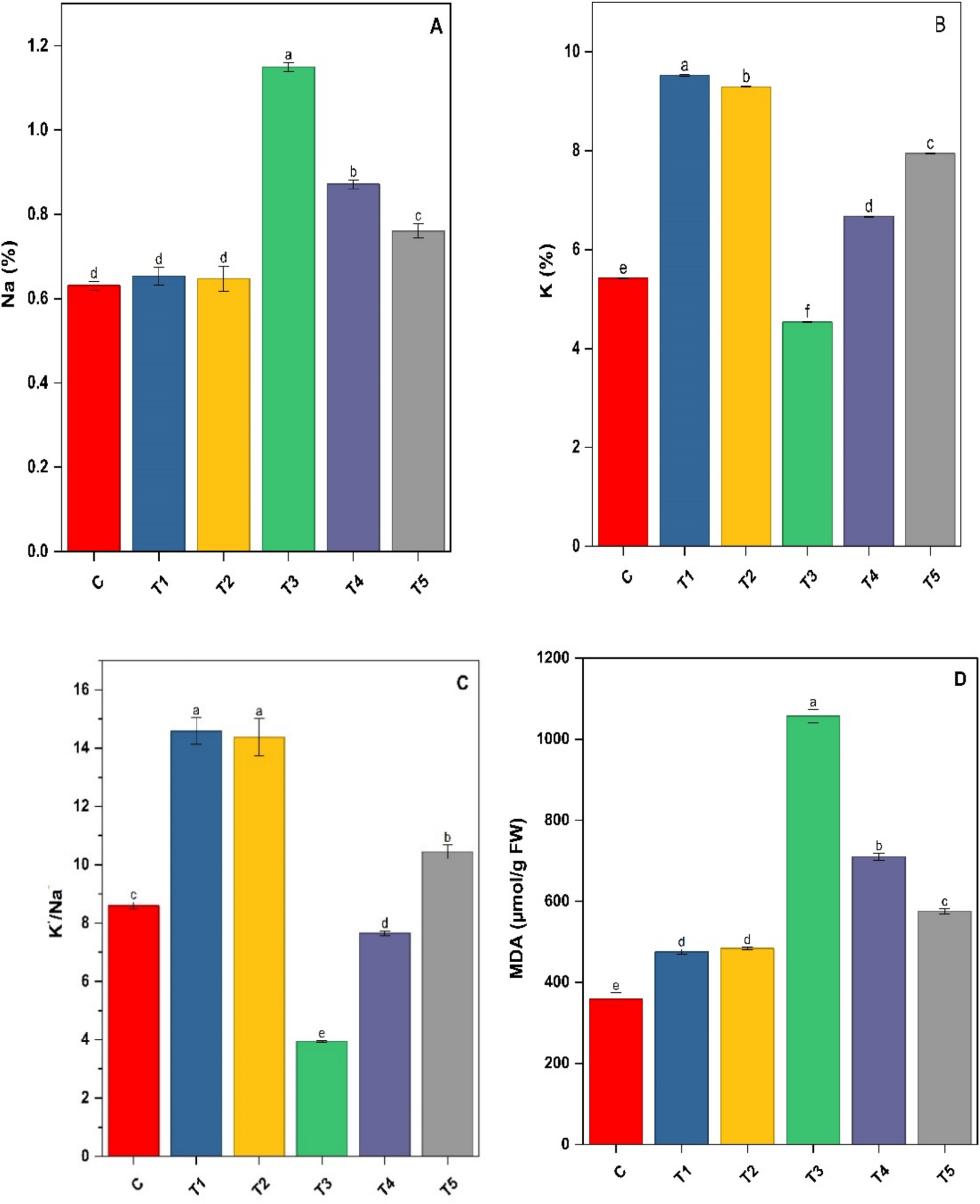
Effect of the biologically synthesized MVCN on sodium, Potassium, potassium sodium ratio and MDA content of sunflower under salt stress stress. (**A**) Na (%), (**B**) K (%), (**C**) K^+^/Na^+^, (**D**) MDA, C = control; T1 = seed priming in 100 mg/L of aqueous solution of MVCN. ; T2 = foliar spraying with 100 mg/L of aqueous solution of MVCN ; T3 = salt stress (150 mM NaCl); T4 = salt stress + seed priming in 100 mg/L of aqueous solution of MVCN; T5 = salt stress+ foliar spraying with 100 mg/L of aqueous solution of MVCN. Different letters on the bars are significantly different as evaluated by Tukey test at a significant level of *p* < 0.05

**Fig. 9:**
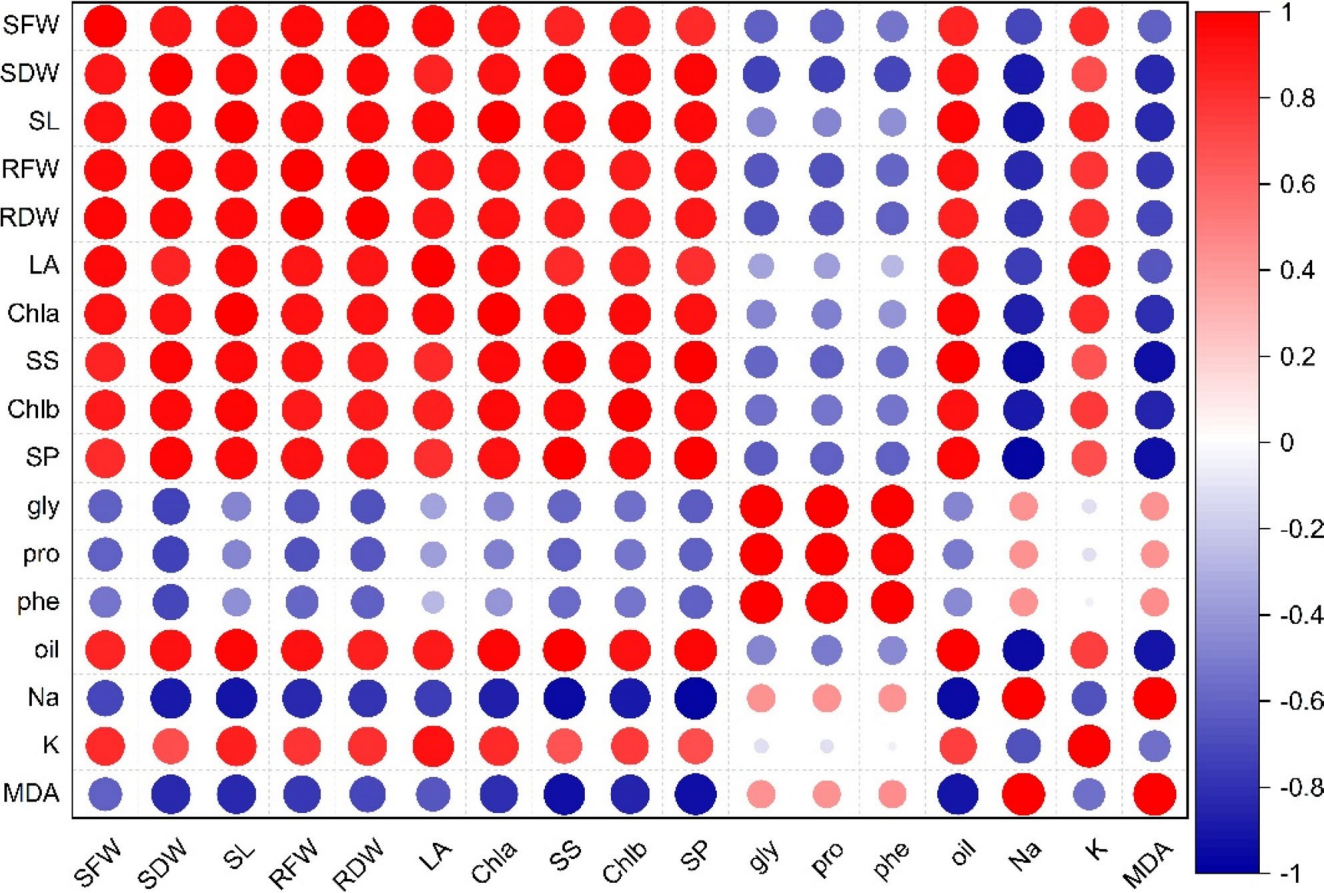
Correlogram based on the correlation coefficients between growth parameters and biochemical traits of sunflower in response to MVCN and salt stress. SFW= shoot fresh weight, SDW= shoot dry weight SL= shoot lenghth, LA= leaf area, Cha= chlorophyll a, Chb= chlorophyll b, SS= soluble sugars, Pro= proline, Phe= phenolics Sp= soluble protein

Exposure of plants to salt stress led to an 82% increase in sodium content. In contrast, the application of nano‑treatment (T5) under salt stress resulted in a 34% decrease in sodium content compared to untreated plants (Fig. 8A). Moreover, nano‑treatments, whether applied through seed priming or foliar spraying, significantly increased potassium (K) content as well as the K/Na ratio in plants, both under salt stress and non‑stress conditions (Fig. 8B & C).Data presented in Fig. 9D showed that malondialdehyde (MDA), a marker of lipid peroxidation, was significantly elevated under salt stress. Specifically, MDA content in salt‑stressed plants increased by nearly 195% compared to control plants. However, nano‑treatment under salt stress reduced MDA levels by 46% relative to untreated plants.

### Correlation analysis

Correlogram based on the correlation coefficients between growth parameters and biochemical traits of sunflower in response to MVCN and salt stress are shown in Fig. 9. The Correlogram demonstrated that all growth parameters, as well as chlorophyll and protein content, were strongly positively correlated with each other. Likewise, a strong positive correlation was observed between phenolic compounds, proline, and glycine betaine content. In contrast, all of these measurements were negatively correlated with sodium content and malondialdehyde levels.

## Discussion

Among the various abiotic stresses, salinity is a major constraint to plant growth and yield, particularly in arid and semi-arid regions [52]. Several strategies have been developed to alleviate salt stress in plants, with the application of nanoparticles being one of the most promising. Extensive research has demonstrated that engineered nanoparticles can stimulate plant growth, enhance crop yield, and improve quality under saline conditions when used in modest dosages [53, 54], . Their use in agriculture also reduces reliance on chemical inputs, minimizes soil and water pollution, and supports the sustainability of crop production systems. Therefore, in the present study, mixed-valence copper oxide (Cu₄O₃) was biologically synthesized, and its potential role in mitigating salt stress in sunflower plants was investigated.

In our earlier article [55], we thoroughly examined the FT-IR spectrum of the *Leucaena leucocephala* seed extract, which showed a number of distinctive absorption bands attributed to different functional groups, such as hydroxyl, carboxyl, amide, and carbonyl groups. Here, the spectrum of biologically synthesized (MVCN) made with L. leucocephala seed extract was shown in Fig. 1 whereas all of the distinctive peaks seen in the plant powder were moved, indicating that the capped MVCN had successfully formed. Additionally, through electrostatic interactions or coordination bonds, functional groups like carboxyl (− COOH), hydroxyl (− OH), and amino (− NH₂) groups interacted with the Cu ions and surfaces of the nanoparticles, stabilizing them and influencing their physicochemical characteristics.The successful creation of Cu_4_O_3_ nanoparticles was confirmed by peaks at 572, 609, and 659 cm^− 1^ in the lower wavenumber area, which may be attributable to metal–oxygen stretching vibrations [56]. The FT-IR results showed that the phytochemicals in the *L. leucocephala* seed extract created a capping layer around the nanoparticles and stabilized the copper ions.

Figure 1 displayes the FTIR spectrum of biologically synthesized MVCN prepared using *L. leucocephala* seed extract Bands of metal–oxygen vibrations may overlap with organic functional groups bands obtained from the extracts [56].The spectrum revealed the existence of a new peak at 3396 cm⁻¹, which ascribed to hydroxyl stretching, indicating the capping efficacy of *L. leucocephala* extract.

The –OH bending frequency of surface hydroxyl groups (moisture), dislayed a peak at 1659 cm^− 1^ [20]. The peak at 2928 cm^− 1^ corresponded to the C–H stretching, the peaks correlated to C–H bending, and C–O bond stretching appeared at 1395 cm^− 1^ and 1109 cm^− 1^ respectively. Further more, functional groups, such as carboxyl (− COOH), hydroxyl (− OH), and amino (− NH₂) groups, interacting with the Cu ions and nanoparticles surfaces via electrostatic interactions or coordination bonds, stabilize the nanoparticles and affect their physicochemical properties. Lower wavenumber region showed peaks at 572, 609, and 659 cm^− 1^ which may attributed to metal–oxygen stretching vibrations confirming the successful formation of Cu_4_O_3_ nanoarticles [57].

Additionally, the electronic absorption spectra of Cu_4_O_3_ nanoparticles exhibited a surface plasmon resonance peak at 475 nm, suggesting Cu_4_O_3_’s inherent band gap absorption produced by electron transitions from the valence to the conduction band [57, 58] The latter absorption peak is different from the absorption peaks of CuO which reported to appear in the range 300–400 nm and Cu₂O which also reported to appear in the range 250–350 nm. This peak agreed with the reported interband transition of parahexachloride (of Cu_4_O_3_, a mixedvalence Cu⁺/Cu^+ 2^ oxide) [59].

The electronic absorption spectrum of the mixed oxide Cu_4_O_3_ measured at room temperature showed the characteristic absorption peak at the wavelength 235and a shoulder at 300 nm. Cu_4_O_3_’s intrinsic band gap absorption as a result of electrons moving from the valence band to the conduction band was identified as the cause of this absorption spectrum pattern [20].

Additionally, the electronic absorption spectra of Cu_4_O_3_ nanoparticles exhibited a surface plasmon resonance peak at 475 nm, suggesting Cu_4_O_3_’s inherent band gap absorption produced by electron transitions from the valence to the conduction band [57, 58, 22]. The latter absorption peak is quite different from the absorption peaks of CuO (300–400 nm) and Cu₂O (250–350 nm). This peak agreed with the reported interband transition of parahexachloride (of Cu_4_O_3_, a mixedvalence Cu⁺/Cu^+ 2^ oxide) [59].

The bio-synthesized MVCN using *Leucaena leucocephala* seeds were characterized via X-ray diffraction, as depicted in Fig. 1C. The diffraction peaks are located at 20^ο^, 25^ο^, 30^ο^, 40^ο^, and 50^ο^ corresponded to (101), (112), (200), (004) and (105) [20]., respectively, which are consistent with JCPDS file No.33–0480 which confirmed the tetragonal copper oxide paramelaconite.

The elemental constituents of the synthesized MVCN was verified by EDX analysis. The detected elements include carbon, oxygen, nitrogen, and iron. The highest weight% of carbon (Wt%) of 38.65%. revealed that, the plant extract used in the biosynthesisof the MVCN is basically composed of sugars, which accounts for the sample’s high carbon and glucose content. The coexistence of oxygen and carbon in the sample suggests that the plant material utilized in the synthesis is predominantly polysaccharidic in nature.

The morphology of the MVCN was characterized by performing a scanning electron microscope (SEM). The synthesized MVCN exhibited a well-separated spherical nanoparticle morphology. The average diameter of the particles, assessed from SEM pictures by arbitrary selection, ranged from 13 to 18 nm, and interconnection between particles existed to a lesser extent. The dispersion stability of the particles in the reaction solution or the precipitation mechanism of the particles may be connected to the tendency for spherical particle formation [60]. In addition, the MVCN effectively suppresses the aggregation and interconnection of the particles and strengthens the structural stability [61].

The transmission electron microscopy (TEM) was also used to characterize the formed Cu4O3 nanoparticles. The mixed-valence copper oxide (Cu_4_O_3_) nanoparticles exhibited a range of mixed morphologies, spherical particles, rod-shaped, and plate-like, which may suggest anisotropic or polymorphic growth during synthesis. Whereas, many synthesis conditions including precursor concentration, nucleation versus growth rate balance and capping/stabilizing agent can produce multiple morphologies [62].

The diameter of rod-shaped particles are within10-16 nm, while the plate shaped particles were in the range of 28–32 nm and the average size of the particles is 30.4 nm ± 1.59. On the other hand the observed spherical-shaped particles showed a length of around 5 nm. The particles were strongly agglomerated, interconnected to each other and few particles were completely separated.

The Zeta potential value verified the stability of the synthesized MVCN. The zeta potential was found to be -19.4 Mv.The growth of OH-groups on the particles’ surface during their dispersion in a water medium is associated with the negative value. Van der Waal’s force causes the dispersed particles in water to agglomerate together.

Additionally, when all of the particles acquire a positive or negative charge on their surface, a repulsion force is created between them.The particles will be more stable and widely distributed if this repulsion force is stronger than Van der Waals’ force. Zeta potential is used to extrapolate this repelling force, and greater stability is correlated with a larger zeta potential [63]. Our particles showed good stability, suggesting their stability in the contexts in which they are used.

Our findings showed that salt stress adversely affected on growth attributes, physiological performance, and biochemical responses. Salt stress negatively influences multiple stages of plant growth and development. It hinders seedling establishment, restricts vegetative growth, disrupts reproductive processes, and ultimately reduces overall crop productivity [64, 65]. It also impacts plants at the cellular level, leading to alterations in ultrastructural components, membrane injury, excessive accumulation of reactive oxygen species, impairment of photosynthetic processes, and diminished enzymatic activity. Collectively, these effects restrict plant development and reduce agricultural productivity [66]. However, foliar application of the biologically synthesized mixed-valence copper oxide in the current stuy alleviated the negative impacts of salt stress on sunflower. Similarly, Hernández-Hernández et al. [66] reported that application of Cu NPs induced tomato growth under salt stress, while Shaikhaldein et al. [25] found that CuNPs positively influenced barley growth under similar conditions. Copper oxide nanoparticles have been explored in agricultural research for their potential role in helping plants cope with salinity stress [67, 52].The improvement in sunflower growth under salt stress after the application of MVCN could be attributed to enhanced mineral nutrient uptake efficiency [68, 69], osmolyte accumulation [69] improvement in gas exchange and optimization of water use efficiency [25].

Alterations in the concentration of photosynthetic pigments are frequently used as indicators of plant tolerance to abiotic stress [70]. Our results indicate that chlorophyll content dramatically decreased in salt-stressed sunflower plants. Similar findings were reported by by Abdel-Fattah et al. [71], Abdelhameed, and Metwally [72] who observed that salt stress negatively affects chlorophyll content. The decline in chlorophyll levels in salt-stressed sunflower plants may be attributed to the oxidative breakdown of chloroplast pigments and the instability of the pigment–protein complex under salinity conditions [73, 74]. Conversely, the results of the current study show that foliar application of biologically synthesized MVCN led to a marked increase in all photosynthetic pigments, including chlorophyll a, chlorophyll b, and carotenoids. This finding is consistent with the observations of Govorov and Carmeli [75], and Osman et al. [76] who reported that metal nanoparticles enhanced photosynthetic pigments and improved photosynthetic efficiency. Furthermore, copper oxide nanoparticles have shown promise in enhancing plant photosynthesis under salt stress by stimulating the activity of key enzymes such as Rubisco and ATPase, thereby supporting improved photosynthetic efficiency [77, 68]. In line with these biochemical improvements, a noticeable enhancement in the leaf ultrastructure of sunflower plants was also observed following nano-treatment. Despite these findings indicating the positive impact of nano treatment on chlorophyll content and photosynthetic activity Pérez-Labrada et al. [78] found that chlorophyll content decreased in tomato plants when treated with copper nanoparticles under salt stress.

Researchers have found that Cu-NPs can activate stress-responsive genes, particularly those involved in the synthesis of osmoprotectants and antioxidants. These compounds are crucial for maintaining cellular homeostasis and stability when plants are exposed to salinity stress [68, 69]. In the present study, plants treated with MVCN exhibited a significant increase in soluble sugars compared with untreated plants under salt stress. Soluble sugars play a pivotal role in mitigating salt stress by scavenging reactive oxygen species (ROS), stabilizing protein structure [79], modulating cellular redox homeostasis [80], and providing energy [81]. The observed increase in soluble sugars in sunflower following nano-treatment can be attributed to copper’s role in carbohydrate biosynthesis. As an essential cofactor, copper (Cu) supports proteins involved in the photosynthetic electron transport chain and facilitates carbon fixation in the Calvin cycle [28]. Conversely, Shaikhaldein et al. [25] reported that the use of CuNPs led to a decrease in sugar content in barley plants under salt stress conditions.

Proline plays an essential role in enhancing salt stress tolerance in plants through multiple protective mechanisms. In sunflower, proline content increased significantly under salt stress, and this effect was further enhanced by the application of biologically synthesized MVCN. Application of copper nanoparticles during salt stress has been shown to enhance proline accumulation [78, 82]. Shadidijazi and Taspinar [83] reported that the observed increase in proline levels may result from upregulated biosynthesis and potentially reduced degradation.Proline mitigates oxidative stress by neutralizing free radicals and reducing reactive oxygen species (ROS), thereby sustaining redox homeostasis and preserving the integrity of membranes and proteins during salt stress [84]. As an osmolyte, proline contributes to maintaining cellular turgor and stabilizing subcellular structures [85]. It also supports ionic homeostasis by reducing Na⁺ and Cl⁻ accumulation while enhancing K⁺ uptake [86]. Furthermore, proline improves photosynthetic performance by increasing chlorophyll content and promoting stomatal conductance [87].

Similarly, the study showed a significant increase in glycine betaine in salt-stressed sunflower plants after treatment with the biologically synthesized MVCN. This compound plays a role in alleviating salt stress by scavenging reactive oxygen species [88], regulating ion uptake and transport [89], and maintaining cellular osmotic balance [90]. Likewise, the application of the biologically synthesized MVCN under salt stress resulted in a significant increase in phenolic content. This behaviour could potentially be attributed to the increased activity of phenyalaine aminolyase (PAL) in response to nano treatment [91] Phenolic compounds play a crucial role in alleviating salt stress in plants through maintaining redox homeostasis [92]. Conversely, Hanif et al. [93] found that the application of ZnO nanocomposite resulted in a decrease in phenolic content of *Coriandrum sativum* Under salt stress.

The present study demonstrated that the use of nanoparticles under salt stress led to a significant improvement in oil content, which had been markedly reduced as a result of salinity stress. In line with our findings, Rameen et al. [94] reported that the application of copper and silver nanoparticles enhanced both the quality and quantity of rapeseed oil. Similarly, Chen et al. [95] observed that metal nanoparticles modulated lipid metabolism–related genes, resulting in alterations that improved the quality of plant-derived oils.

Salinity stress induces both osmotic imbalance and ion toxicity by promoting sodium accumulation and reducing the K⁺/Na⁺ ratio, largely due to diminished osmotic potential in plant roots [52]. Under such conditions, sodium ions compete with potassium, leading to potassium deficiency [96]. These ionic disruptions interfere the uptake and distribution of essential nutrients, thereby disturbing key physiological processes in plants [97]. Consistent with this context, the present study demonstrated that Na (%) increased significantly, whereas K (%) and the K⁺/Na⁺ ratio declined in plants exposed to salt stress compared to controls. Notably, application of the biologically synthesized MVCN under salt stress resulted in a significant reduction in Na (%) and an improvement in the K⁺/Na⁺ ratio. One possible explanation of this is the effect of nanoparticles on membrane transport throught enhancing the activity of Na+/H+ antiporter), which promoting Na+ efflux and reduce cytoplasmic Na+ accumulation [98]. Another mechanism is that nanoparticles could increase the NHX expression as Na+/H+ exchanger (NHX) transports Na+ from cytoplasm into vacuoles, sequestering toxic ions [99]. Thus, maintaining ion homeostasis emerges as a critical factor in determining plant salt tolerance [96].

In the current study, the level of MDA drastically increased in sunflower plants in response to salt stress. This pronounced rise in MDA levels can be attributed to the oxidative stress and the reduction in the water status of stressed plants, which results from the excessive accumulation of sodium ions [100, 101]. The malondialdehyde (MDA) content in sunflower plants decreased significantly under salt stress following the application of biologically synthesized MVCN, compared to untreated salt-stressed plants.This reduction in MDA levels suggests that the detrimental effects of salinity on sunflower were substantially alleviated. Such mitigation may be attributed to the influence of copper nanoparticles on the antioxidant system. Pérez-Labrada et al. [78] reported that applying copper nanoparticles as a foliar treatment to tomato plants under salt stress led to a rise in superoxide dismutase (SOD) activity and attributed this enhancement to the induction of SOD expression, as copper nanoparticles provide bioavailable copper required as cofactors for SOD. Similarly, Li et al. [102] reported that nanoparticle treatments favorably modulated the ascorbate–glutathione system. In addition, the significant increase in osmoprotectants (proline, phenols, and soluble sugars) in salt-stressed sunflower after the application of the biologically synthesized MVCN could explain the mitigation of oxidative stress, as these osmoprotectants are known for their role in scavenging reactive oxygen species [103, 78, 84].

In addition to the aforementioned mechanisms, a possible explanation for the role of biologically synthesized mixed-valence copper oxide (Cu₄O₃) in improving sunflower performance under salinity stress may be linked to the critical role of copper in plant physiology. Copper participates in enzymatic systems that regulate diverse biochemical reactions and is fundamental to photosynthesis, protein and carbohydrate metabolism, respiration, and overall growth and development [107]. At the nanoscale, copper further provides agronomic advantages, including enhanced bioavailability, sustained nutrient release, and precise cellular-level delivery, thereby offering superior efficacy compared to conventional fertilizer formulations [30].

The current study demonstrated that foliar application of the biologically synthesized MVCN had a superior effect compared to seed priming in mitigating the impact of salinity on sunflower plants. Similarly, Hong et al. [104] reported that foliar application of nanoparticles produces faster and better outcomes than soil application.This advantage can be attributed to direct uptake through stomata and cracks on the leaf surface, as well as higher bioavailability [105]. Moreover, the interactions between nanoparticles and biomolecules may elicit stress-responsive pathways, leading to the accumulation of osmoprotectants and antioxidants [106].

The advanced techniques employed in this study to characterize the biologically synthesized MVCN revealed that the nanoparticles possessed a size range of 13–18 nm. Their nanoscale dimensions and large surface area enable them to readily penetrate cellular barriers through plasmodesmata pore formation, endocytosis, and transport proteins, as well as interact with intracellular structures [108, 109]. Notably, the size of MVCN falls within the optimal range known to promote plant growth and development [110]. FTIR spectroscopy confirmed the presence of functional groups that interact with MVCN. These interactions contribute to the stabilization of MVCN and enhance its bioavailability [111]. The XRD analysis of the biologically synthesized MVCN revealed crystalline structure (tetragonal copper oxide paramelaconite). The nanoscale dimensions and crystalline architecture of the biologically synthesized MVCN provide these materials with elevated surface energy and reactivity, characteristics that are essential for their assimilation into plant metabolic pathways during abiotic stress adaptation [112] Zeta potential analysis provided additional confirmation of the stability of the biologically synthesizes MVCN. Beyond these physicochemical properties, MVCN also offer a highly important economic advantage: nanoparticles have the potential to induce systemic resistance in plants, thereby providing protection against pathogenic attacks [113]. Overall, the unique physical and chemical properties of biologically synthesized MVCN suggest that they may play a significant role in activating stress-responsive pathways, ultimately leading to increased osmolyte accumulation as a defense mechanism during salt stress.

The present study demonstrates that *Leucaena leucocephala* seed extract can serve as an effective and sustainable reducing agent for the biological synthesis of mixed‑valence copper oxide (Cu₄O₃) nanoparticles. Unlike previous studies that required prolonged reflux at elevated temperatures (e.g., 97–100 °C for several hours) using Razma seed extract [20], pumpkin seed extract [31], or Aegle marmelos leaf extract [22], our approach achieved nanoparticle formation under milder conditions simply by mixing a 1:2 (v/v) ratio of 3 mM CuSO₄·5 H₂O with the seed extract. The immediate color change from blue to green provided a visual indicator of successful synthesis, highlighting the efficiency of this method. This simplified protocol not only reduces energy consumption but also enhances cost‑effectiveness and accessibility. Furthermore, while earlier studies have employed organic solvents such as N, N‑dimethylformamide (DMF) and ethanol [21], our use of distilled water as the reaction medium underscores the environmental compatibility of the process. By eliminating hazardous solvents, the method aligns with principles of green chemistry and offers long‑term ecological safety. Collectively, these features establish the novelty of our synthesis strategy and reinforce its sustainability.

## Limitations of the study and future research directions

One important limitation of this study is the potential phytotoxic effects of biologically synthesized MVCN when applied at higher concentrations. Another limitation involves the limited understanding of the environmental fate and potential risks associated with MVCN. Future research should aim to elucidate uptake pathways, evaluate long‑term stability within plant tissues, investigate translocation across trophic levels, and assess persistence in agroecosystems. Addressing these gaps is crucial to ensure the safe and sustainable application of nanotechnology in agriculture, particularly in the context of climate change challenges. Moreover, future research should focus on integrating MVCN applications with other abiotic stress alleviation strategies, such as biofertilizers and plant growth-promoting rhizobacteria (PGPR). In addition, molecular investigations are required to better understand the mechanisms through which MVCN regulates stress tolerance. Advanced genomic and transcriptomic approaches could help elucidate the interactions between MVCN and stress-responsive signaling networks. Such combined approaches could enhance plant resilience and support sustainable crop productivity under adverse environmental conditions.

## Practical implications and scalability

The promising results of biosynthesized mixed-valence copper oxide (Cu₄O₃) nanoparticles (MVCN) in mitigating salinity stress highlight their potential for agricultural application; however, translation to field conditions requires further validation across diverse soils, climates, and salinity levels. Foliar spraying, which proved more effective than seed priming, offers practical compatibility with existing farming practices. While the biosynthesis method using Leucaena leucocephala seed extract is cost-effective and eco-friendly, large-scale production demands careful evaluation of raw material availability, extraction efficiency, and nanoparticle yield. Moreover, optimization of dosage, application frequency, and delivery methods will be critical for scalability, alongside long-term assessments of environmental safety. Successful adoption will also depend on navigating regulatory frameworks governing nanomaterials in agriculture, necessitating collaboration among researchers, agronomists, policymakers, and industry stakeholders to ensure both efficacy and compliance.

## Conclusion

This study establishes a simple, eco‑friendly, and cost‑effective strategy for the sustainable synthesis of mixed‑valence copper oxide (Cu₄O₃) nanoparticles using *Leucaena leucocephala* seed extract, which acts as a natural reducing, stabilizing, and capping agent. Characterization by EDX, XRD, FTIR, UV–Vis, SEM, TEM, and Zeta potential analyses confirmed the successful synthesis of Cu₄O₃ nanoparticles. FTIR analysis verified effective capping by seed extract, while SEM revealed spherical particles (13–18 nm) and TEM indicated diverse morphologies, suggesting anisotropic growth. The biologically synthesized MVCN, particularly when applied as foliar sprays (100 mg/L), significantly enhanced sunflower growth and improved key biochemical traits, while reducing sodium accumulation and oxidative damage. These outcomes illustrate the potential of MVCN as a sustainable nanotechnology‑based strategy to enhance crop resilience and productivity under saline conditions, with promising implications for agriculture in the face of climate change.

## Supplementary Information

Supplementary Material 1.

## Data Availability

All data generated or analyzed during this study are included in the current article.
