# Supplementary material for: Seed priming versus foliar spraying of biologically synthesized mixed-valence copper oxide(Cu4O3): improving sunflower growth under salt stress
Source: BMC Plant Biol. 2026 Jul 1;26:1112. doi: 10.1186/s12870-026-09334-8 (PMC13326334; doi:10.1186/s12870-026-09334-8)
Supplement: Supplementary file 1 — Supplementary Material 1. [file 12870_2026_9334_MOESM1_ESM.docx]

**1-Physical and Chemical Characterization of the biosynthesized MVCN**

**1.1.-ray photoelectron spectroscopy**

Thermo Fisher Scientific, USA's K-ALPHA was used to perform X-ray photoelectron spectroscopy (XPS) investigations using monochromatic X-ray Al K-alpha radiation (energy -10 to 1350 eV) in a vacuum of 10-9 mbar at full-spectrum pass energy of 200 eV and narrow-spectrum 50 eV. The diameter of the analysis point was 400 μm. Every binding energy value was calculated with reference to the adventitious carbon-derived C1s line at Central Metallurgical Research, Institute (CMRDI), Al Tbin, Cairo, Egypt.

**1.2. Fourier transform infrared spectroscopy**

The identification of probable biomolecule in charge of the formation of a binary FeOOH/Fe2O3 nanosized composite was accomplished by FTIR analysis. The BFNC and potassium bromide (KBr) were combined in a hydraulic press to create the sample, which was subsequently dried to eliminate any remaining moisture. An FTIR spectrophotometer (FTIR spectrum Version 10.5.3, Perkin Elmer) was used to record infrared spectra across a range of 450–4000 cm–1.

**1.3. Energy-dispersive X-ray analysis**

Using an X-ray micro-analyzer (Module Oxford 6587INCA X-sight) in conjunction with a scanning electron microscope (SEM-IT 200; JOEL Corp, Tokyo, Japan) operating at 20 kV of an accelerating voltage for compositional analysis and elemental Fe confirmation, the structure of binary FeOOH/Fe2O3 nanosized composite was described by an energy-dispersive X-ray analysis (EDX) spectrum.

**1.4.Scanning electron microscopy**

The SEM JEOL IT 200 (JOEL Corp, Tokyo, Japan) was used to determine the size and form of the green synthesized BFNC. After combining the biosynthesized powder with double-distilled water, it was sonicated for half an hour. On a glass slide, a tiny drop of this material was let to dry, creating a thin coating of NPs.

**1.5.Transmission electron microscopy**

A TEM JEM-1400 plus transmission electron microscope (JOEL Corp, Tokyo, Japan) running at an accelerating voltage of 50 kV was used to investigate the size and morphology of the BFNC. A drop of BFNC solution was applied on a copper grid coated with carbon, and water evaporation ensued.

**1.6. X-ray diffraction**

X-ray diffraction was conducted utilizing a Phaser XRD-D2 powder X-ray diffractometer (Bruker, Germany) equipped with Cu-Kα radiation throughout a broad spectrum of Bragg angles θ (10° ≤ 2θ ≤ 80°), functioning at 30 kV and 10 mA. Data were compared to the Joint Committee on Powder Diffraction Standards' published standard data (JCPDS Card No. 36–1451). Debye-Scherrer's equation was used to interpret the crystallite size (nm) from the XRD line broadening measurement: (D = λk/β cosθ), where θ is the diffraction angle, λ is the wavelength of X-ray radiation, λ = 1.5406 Å, and β is the full width at half maximum (FWHM) of the most intense diffraction peak.

1.7. **UV–Visible spectroscopy**

The biosynthesized BFNC was characterized using UV–Vis spectrophotometer (Thermo Scientific Evolution TM 300) in the wavelength region between 200-nm and 800-nm, operated at a resolution of 1 nm, and maximum absorbance was determined.

1.8. **Zeta potential measuremen**t

One milligram of BFNC was dissolved in ten milliliters of distilled water, sonicated for five minutes, and then measured with a Malvern Zetasizer Nano ZS analyzer (Malvern Instruments, Malvern, UK)66 to determine the zeta potential.

**2. Determination of photosynthetic pigments**

The chlorophyll a , (Cha ), chlorophyll b (Chb) were measured spectrophotometrically at 647 and 664 nm following N, N-dimethylformamide (DMF) method as described by Inskeep and Bloom [47]. The following formula and extinction coefficients were utilized to determine the the concentrations of the  photosynthetic pigments:

Chla = 12.70 A_665_ – 2.79 A_647_

Chl.b = 20.70 A_647_ – 4.62 A_665_
